# Left Ventricular Function Evaluation on a 3T MR Scanner with Parallel RF Transmission Technique: Prospective Comparison of Cine Sequences Acquired before and after Gadolinium Injection

**DOI:** 10.1371/journal.pone.0163503

**Published:** 2016-09-26

**Authors:** Thibault Caspar, Anthony Schultz, Mickaël Schaeffer, Aïssam Labani, Mi-Young Jeung, Paul Thomas Jurgens, Soraya El Ghannudi, Catherine Roy, Mickaël Ohana

**Affiliations:** 1 Cardiology Department, Nouvel Hôpital Civil, Strasbourg University Hospital, Strasbourg, France; 2 Radiology Department, Nouvel Hôpital Civil, Strasbourg University Hospital, Strasbourg, France; 3 Public Health and Biostatistics Department, Nouvel Hôpital Civil, Strasbourg University Hospital, Strasbourg, France; 4 University of Colorado, Denver, Colorado, United States of America; 5 iCube Laboratory, Université de Strasbourg / CNRS, UMR 7357, 67400, Illkirch, France; Worcester Polytechnic Institute, UNITED STATES

## Abstract

**Objectives:**

To compare cine MR b-TFE sequences acquired before and after gadolinium injection, on a 3T scanner with a parallel RF transmission technique in order to potentially improve scanning time efficiency when evaluating LV function.

**Methods:**

25 consecutive patients scheduled for a cardiac MRI were prospectively included and had their b-TFE cine sequences acquired before and right after gadobutrol injection. Images were assessed qualitatively (overall image quality, LV edge sharpness, artifacts and LV wall motion) and quantitatively with measurement of LVEF, LV mass, and telediastolic volume and contrast-to-noise ratio (CNR) between the myocardium and the cardiac chamber. Statistical analysis was conducted using a Bayesian paradigm.

**Results:**

No difference was found before or after injection for the LVEF, LV mass and telediastolic volume evaluations. Overall image quality and CNR were significantly lower after injection (estimated coefficient cine after > cine before gadolinium: -1.75 CI = [-3.78;-0.0305], prob(coef>0) = 0% and -0.23 CI = [-0.49;0.04], prob(coef>0) = 4%) respectively), but this decrease did not affect the visual assessment of LV wall motion (cine after > cine before gadolinium: -1.46 CI = [-4.72;1.13], prob(coef>0) = 15%).

**Conclusions:**

In 3T cardiac MRI acquired with parallel RF transmission technique, qualitative and quantitative assessment of LV function can reliably be performed with cine sequences acquired after gadolinium injection, despite a significant decrease in the CNR and the overall image quality.

## Introduction

MRI is currently the standard technique for the assessment of cardiac chambers volumes and function [1,2], with the best reproducibility [3]. Cardiac MRI (CMR) is particularly useful when a serial follow-up of left ventricular (LV) function is anticipated, with the need for accurate and reproducible quantification such as when monitoring the cardiotoxic effects of cancer chemotherapy [4]. While the list of clinical indications is consistently increasing [5], MR accessibility remains limited by the availability of scanners and by time-consuming examinations. Consequently, there is a strong interest in optimizing the cardiac MRI acquisition protocols in order to shorten them as much as reasonably achievable [6–8], and a push towards utilizing all available MR resources, whether it be 1.5T or 3T scanners [9–12].

In addition to these cine sequences dedicated to the evaluation of the cardiac chamber volumes and ventricular systolic function, CMR protocols frequently include late-gadolinium enhancement (LGE) sequences that are acquired 5 to 10 minutes after the contrast agent injection [13,14]. To guarantee optimal image quality and maximum contrast between the blood pool and the myocardium, it is recommended to acquire the cine sequences before the gadolinium injection, thus leading to a « dead time » interval in the CMR protocol between the contrast media injection and the LGE sequences. It has already been suggested to take advantage of this impregnation time to acquire the cine images, so as to shorten the total examination time. If at 1.5T, some authors [15,16] have demonstrated that LV delineation remains accurate enough to correctly assess wall motion and LVEF, it has not yet been proven that b-TFE cine sequences obtained at 3T could be acquired after gadolinium injection, all the more since the difference in T1 between the blood pool and the myocardium is decreased when increasing the field strength [16]. If post-contrast is currently a common practice in many 1.5T CMR sites, its generalization to 3T sites is not yet supported by the current literature.

The aim of this study is therefore to compare the diagnostic performances and the image quality of CMR cine sequences acquired before and after gadolinium injection on a 3T MR scanner equipped with dual-source RF transmission technology.

## Material and Methods

This study was approved by our institutional review board (Comité de Protection des Personnes Est—IV) and written informed consent was obtained from all participants.

### Study population

For 2 consecutive months (July and August 2012), all patients referred to our department for a gadolinium-enhanced CMR were prospectively enrolled. Exclusion criteria were, in addition to the classical contraindications to MRI or to gadolinium injection (*i*.*e*. severe renal impairment with an eGFR<30 mL/min, proven allergy to gadolinium), a subject younger than 18 years old, a pregnancy, inability to give informed consent, or a severe agitation or any other condition that could interfere with the patient’s ability to comply with the examination.

### MR Imaging

All patients underwent a 3T cardiac MRI (*Achieva 3*.*0T X-series*, Philips Medical Systems, Best, The Netherlands) equipped with a fully flexible dual-source RF transmission technology (*i*.*e*. Tx MultiTransmit^®^) and a dedicated 6-channel SENSE torso coil.

The same acquisition protocol was applied to all patients. Morphological « Black-Blood » T1 and T2-weighted Spin Echo Single Shot sequences were first acquired. Then the ECG-gated cine B-TFE (« *Balanced Turbo Field Echo* ») sequences were acquired in the three cardiac planes (LV short- and long-axis, 4-chamber) in two steps: before contrast agent injection and immediately following the injection of a gadolinium-based contrast agent (*Gadovist*, Bayer Healthcare, Leverkusen, Germany) at a dose of 0.1 mmol/kg (flow rate 3mL/s), followed by a saline flush of 30mL (flow rate 3mL/s). Acquisition parameters of the b-TFE cine sequences in the LV short axis plane are summarized in Table 1. Only one slice was acquired for the long-axis as well as for the 4-chamber view, with the same acquisition parameters as for the short-axis. The imaging protocol ended with LGE sequences: 3D Inversion-Recovery (*3D-IR*), acquired at 9 minutes after a Look-Locker sequence to visually determine the inversion time and *3D-PSIR* acquired 13 minutes after the injection. The T1 and T2 black blood sequences and the LGE sequences were only used for clinical purpose, and their quantification was out of the scope of this study.

**Table 1:** Acquisition parameters of the b-TFE cine sequences in the LV short axis plane.

	Cine b-TFE
**echo time**	1.3 to 1.6 ms
**repetition time**	2.7 to 3.2 ms
**flip angle**	45°
**acquisition resolution**	2 x 1.6 mm
**reconstructed resolution**	1.15 x 1.15 mm
**slice thickness acquisition**	8 mm
**number of phases per slice**	30
**acquisition time per slice**	15 sec
**number of slices**	6 to 9
**approximate acquisition time**	3 min 45 sec

### Qualitative evaluation of cine sequences

All cine b-TFE sequences were independently and blindly evaluated in a randomized order by two chest radiologists (AS and MO, with 2 and 5 years of experience in CMR respectively). Qualitative assessment of the cine images in the three cardiac planes was performed on a dedicated workstation (MR Viewforum in version 2.6.3.4, Philips Medical Systems, Best, The Netherlands) using a 4-level Likert scale successively evaluating the overall image quality, the edge sharpness of the cardiac chambers (qualitative evaluation only), the artifacts and the visual assessment of left ventricular wall motion (Table 2). One score was given for each item by evaluating all the short-axis slices, the long-axis and the four-chamber view. There were in total 8 ratings per patient, with 4 points assessed before and 4 after the gadolinium injection.

**Table 2:** Qualitative 4-level scale.

	Overall image quality	Edge sharpness of the cardiac chambers	Artifacts	Visual appreciation of LV wall motion
**1**	Insufficient	Indistinguishable	Severe, interfering with the evaluation	Impossible
**2**	Poor	Blurry	Moderate, partially interfering	Insufficient
**3**	Fair	Visible	Mild, not interfering	Correct
**4**	Excellent	Sharp	Minimum to no artifact	Optimal

### Quantitative evaluation of cine sequences

The quantitative assessment was blindly performed by a single observer (AS) on the cine b-TFE sequences in the LV short axis after randomization of patients as well as pre- and post-contrast images. LV ejection fraction, LV end-diastolic volume and LV myocardial mass were measured using dedicated software (*MR Viewforum* in version 2.6.3.4, Philips Medical Systems, Best, The Netherlands) with manual delineation of the LV epicardium and endocardium on the telesystolic and telediastolic phases, after optimal adaptation of the window settings so as to get an optimal and reproducible visualization of the LV myocardium.

**Fig 1:**
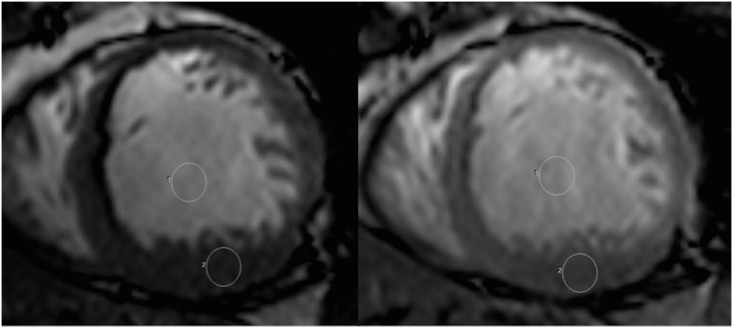
Placement of the ROIs in the left ventricular short axis before and after gadolinium injection.

Contrast to noise ratio (CNR) was obtained by the signal intensity measurement of the LV cavity and of the adjacent healthy myocardium in the LV short axis, with a circular region of interest (ROI) averaging at least 0.5 cm^2^ (Fig 1) using the following formula:

$$CNR=\frac{LV\;CAVITY\;SIGNAL-MYOCARDIUM\;SIGNAL}{\frac{LV\;CAVITY\;SD+MYOCARDIUM\;SD}{2}}$$

This ROI was positioned in the same region (basal inferior wall) in each patient, with careful exclusion of off-resonance artifacts. CNR was measured on a single (end-diastolic) temporal phase of all the LV short axis slices.

### Statistical analysis

Descriptive analyses were calculated by using generic statistics, such as mean, standard deviation, min, and max for quantitative variables. Effectives and percentages were used for qualitative variables. Cohen’s Kappa with quadratic ponderation was used to measure concordance between readers.

Inferential analyses were calculated utilizing a Bayesian paradigm [17–19], by using MCMC methods to evaluate posterior distributions and to measure the probability for a coefficient to be positive. MCMC methods are based upon simulations under specific assumptions, and empirical posterior distributions are evaluated for concludes. Quantitative evaluation scores on a scale of 4 levels were grouped into two categories, 0 for levels 1 and 2 (insufficient quality), and 1 for levels 3 and 4 (sufficient diagnostic quality). Then, comparisons between sequences were made using hierarchical logistic regression models in order to take into account two random effects and thus intraclass variability: the reader and the subject effect. Posterior mean and 95% confidence intervals were then estimated. Coefficients in which the probability of being positive was either more than 97.5% or less than 2.5% were considered as non-zero. This is equivalent to verifying if the 95% credibility interval was different from 0 or not.

Analysis was performed on both software WinBUGS version 1.4.3. (Windows Bayesian Inference Using Gibbs Sampling) and R version 3.0.1 (R Foundation for Statistical Computing, Vienna, Austria).

## Results

### Study population

25 consecutive patients (mean age of 55 ± 14 years-old, minimum 18 yo and maximum 77 yo; 64% male) were ultimately enrolled in this study. 5 (20%) patients had signs of ischemic cardiomyopathy, 1 patient had an acute myocarditis and 6 (24%) were diagnosed with idiopathic (hypertrophic or dilated) cardiomyopathy. The remaining 8 examinations were normal. LVEF was altered (<55%) in 9 (36%) patients and normal in 16 (64%). LV end-diastolic volume was increased in 8 patients (32%), and LV mass was increased in 3 (12%) patients. Regarding LV wall motion, 14 patients (56%) had normal wall motion, whereas segmental hypokinesia was present in 11 patients (44%), segmental akinesia in 2 patients (8%) and segmental dyskinesia in 2 patients (8%).

### Qualitative evaluation

**Fig 2:**
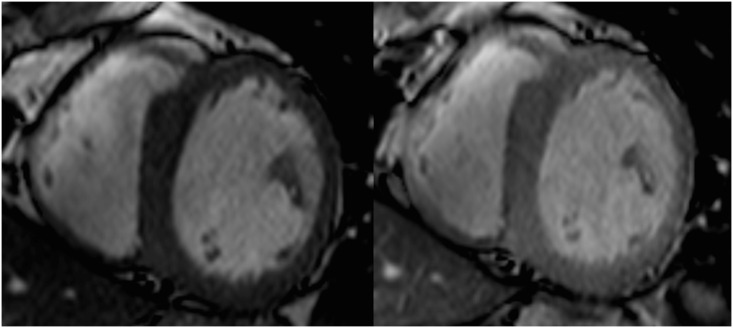
LV short axis cine b-TFE slice in the telediastolic phase before (left) and after (right) gadolinium contrast injection.

Descriptive analysis revealed quantitative superiority of the cine sequences acquired before contrast agent injection for all criteria (Fig 2, Table 3).

**Table 3:** Results of the descriptive analysis for qualitative evaluation.

	Cine before injection	Cine after injection
**Overall quality**	3.54±0.61	3.22±0.84
**Edge sharpness**	3.64±0.60	3.12±0.74
**Artifacts**	3.34±0.69	3.14±0.62
**LV wall motion**	3.8±0.40	3.6±0.57

Bayesian analysis (Table 4) confirmed the statistical significance for overall image quality and edge sharpness. The same trend was observed for the artifacts (dark banding, bright banding or flow off-resonance artifacts) and the evaluation of LV wall motion yet without reaching statistical significance.

**Table 4:** Results of the inferential bayesian analysis for the qualitative evaluation.

**cine after injection > cine before injection**		**Overall Image Quality**	**Artifacts**	**Edge sharpness**	**LV wall motion**
	**Mean of difference**	-1.7463	-0.0399	-2.1224	-1.4670
	**Credible interval [2.5; 97.5]**	[-3.7860; -0.0305]	[-0.1187; 0.0390]	[-4.2950; -0.2973]	[-4.7180; 1.1271]
	**Likelihood**	0.0228*	0.1549	0.0092*	0.1483

* means statistical significance.

### Quantitative evaluation

There was no significant difference in terms of LVEF, LV end-diastolic volume and LV myocardial mass measured before and after gadolinium injection. This was the case whether the LVEF was normal (16 patients) or altered (9 patients). Mean intra-patient variability for LVEF was only 5.1% ± 4.2 (minimum 0% and maximum 13%). CNR was significantly inferior after gadolinium injection (mean drop of 23%).

The results of the descriptive and bayesian analyses are reported in Tables 5 and 6 respectively.

**Table 5:** Results of the descriptive analysis for the quantitative evaluation.

	Cine before injection	Cine after injection
**LVEF (%)**	52.6 ±16.4	52.2 ±16.9
**Intra-patient variability for LVEF (min—max)**	5.1% ±4.2 (0–13)	
**End-diastolic volume (mL)**	162.8 ±60.7	161.8 ±57.7
**Intra-patient variability for End-diastolic volume (min—max)**	5.6% ±4.5 (0–18)	
**LV Mass (g)**	128.2 ±46.8	130.8 ±42.6
**Intra-patient variability for LV Mass (min—max)**	9.4% ±7.3 (0–26)	
**CNR**	19.7 ±13.1	15.2 ±7.3
**Intra-patient variability for CNR (min—max)**	33.5% ±23.7 (1–88)	

**Table 6:** Results of inferential bayesian analysis for quantitative evaluation.

**Cine after > cine before**		**LVEF**	**EDV**	**LV Mass**	**CNR**
	**Mean of difference**	-0.3230	-0.0032	2.5500	-0.2260
	**Credible interval [2.5; 97.5]**	[-3.7860; 0.9815]	[-0.0645; 0.0584]	[-1.6250; 10.9400]	[-0.4930; 0.0425]
	**Likelihood**	0.3072	0.4604	0.7319	0.0473*

* means statistical significance.

### Inter-reader agreement

Measurement of Cohen’s Kappa (Table 7) found mild to moderate agreement between the two readers for the evaluation of overall image quality, edge sharpness and appreciation of LV wall motion. There was a high degree of agreement in the assessment of artifacts. Agreement between the two readers was lower for all criteria in the sequences acquired after gadolinium injection, except for the evaluation of LV wall motion.

**Table 7:** Cohen’s Kappa with quadratic ponderation.

	Cine before	Cine after
**Overall quality**	0.40 ±0.17	<0.2
**Edge sharpness**	0.66 ±0.37	0.25 ±0.33
**Artifacts**	0.79 ±0.09	0.59 ±0.18
**LV wall motion**	0.31 ±0.08	0.38 ±0.05

## Discussion

This work answered our initial question: despite significantly worsened overall image quality, the b-TFE cine sequences acquired at 3T after gadolinium injection are as effective as those acquired before in terms of quantitative (ejection fraction, volume and mass) and qualitative (wall motion) assessment of the left ventricle.

The acquisition of these cine sequences right after the gadolinium injection, during the impregnation time, shortens the examination by about 3 to 4 minutes. This can be added with other optimizations in the choice of LGE sequences [20] to significantly decrease the total examination time. In our university hospital, implementation of these optimizations (both for LGE and cine sequences) has enabled us to increase our number of CMR examinations by around 30%.

Despite wide utilization in the routine clinical setting, very few studies investigating the diagnostic performances of post-contrast cine sequences exist in the literature. Their conclusions are in agreement with ours, even though they were conducted using other contrast agents and/or other sequences than the ones used in the present work. At 1.5T, two studies showed no difference in the overall image quality [15] or in the evaluation of global and regional LV function [15,21] between SSFP cine sequences acquired before and after gadolinium contrast agent. Gerretsen et al. [22] demonstrated that gradient echo cardiac cine imaging at 3.0 T after injection of the intravascular agent gadofosveset leads to improved objective and subjective cardiac cine image quality as well as the same conclusions regarding cardiac ejection fraction compared to balanced steady state free precession (bSSFP) imaging at 1.5 T. This blood pool contrast agent is however not comparable to gadolinium and cannot be used for delayed-enhancement cardiac imaging. At 3T, Hamdan et al. [23] suggested that the use of an extracellular contrast agent (gadopentate dimeglumine) improves the image quality for the assessment of LV volumes for turbo gradient echo sequence in the long axis but not in the short axis. After the injection, LVEF seemed underestimated with the long axis slices, whereas it was overestimated in the short axis views. The same author showed a strong correlation for LV volumes and EF between conventional turbo gradient echo imaging before contrast injection and k-t BLAST (k-space over time broad-use linear acquisition speed-up technique) sequence acquired immediately after administration of gadobenate dimeglumine [24]. These two works at 3T, in addition to using research sequences that are not routinely available, do not reach clear usable conclusions.

The presence of gadolinium both in the blood pool and in the myocardial interstitium is responsible for the CNR decrease and thus the decrease of the LV edge sharpness definition and the overall image quality, in comparison to the cine sequence acquired before injection.

Indeed, myocardial signal increases more than the intracavity signal (+53% versus +3%, in our study), thus leading to both signal values being closer and to a 23% drop of the CNR. This has been studied more formally on a small cohort by Sharma et al [16], with an absolute difference of T1 between the blood pool and the myocardium of 0.53sec before and 0.16sec after the gadolinium injection at 1.5T, and a difference of 0.39sec before and 0.14sec after at 3T. In fine, and despite this qualitative loss that surprisingly wasn’t found on 1.5T studies, the assessment of the LV EF, LV volume and LV myocardial mass remains highly reliable on the post-contrast cine sequences.

In our study, the mean intra-patient differences for the LVEF, the LV volume and the LV myocardial mass were statistically non-significant based on the bayesian analysis. They are in agreement with the intra-observer variability described in the literature for CMR and superior to the intra- and inter-observer reproducibility obtained by other cardiac imaging modalities [25]. Indeed, MRI performances in terms of reproducibility for the measurement of volumes and LVEF are excellent, and can be optimized by the experience of the reader (variability of LVEF improved from 7.2 to 3.7% after two month-experience in a work conducted by Karamitsos et al. [26]). The inter- and intra-observer variability for the estimation of LVEF in echocardiography is higher at around 10% for the biplane Simpson reference method [27]. Using a fully manual software, with epicardium and endocardium delimitation manually delineated by the reader, probably facilitated this result. Even though LV edge sharpness is of lower quality after gadolinium injection, the visual qualitative visualization of left ventricular wall motion is not affected by the gadolinium injection. On this representative population sample with normal and abnormal LV wall motion (hypokinesia, dyskinesia as well as akinesia), the assessment of the LV regional systolic dysfunction was *in fine* as good as in the sequences before gadolinium injection, meaning that the reader could compensate the loss in the overall image quality and perfectly identify the LV margins. That is certainly why the quantitative manual LV evaluation is identical between both sequences; we can speculate that such consistent results would not have been obtained with fully automatic LV quantification software, for which the image quality and CNR loss could lead to incorrect segmentation.

The use of a dual-source RF transmission technology could have helped in compensating some of the usual pitfalls of 3T CMR. This technology improves the performance of MR high-field imaging by reducing dielectric shading, B1 inhomogeneity and local energy deposition, and therefore enhances the image quality and reduces the acquisition time [28]. Advantages of dual-source parallel RF transmission have first been evaluated for MR imaging of the abdomen, pelvis [29] and spine [30]. More recently, CMR studies have shown that the implementation of RF shimming improves image homogeneity, CNR and diagnostic confidence [31–34]. The use of this technology in the present study has probably limited the deterioration of the image quality after gadolinium injection, with fewer off-resonance artifacts, whose presence is closely related to the non-uniformity of the RF field.

This study has several limitations. One of the study limitations is inherent in the Bayesian inference analysis: although it enables superior statistic power with simulation of large population derived from our study sample, a binary clustering of all qualitative data was needed (class 1 and 2 grouped in category 0, class 3 and 4 grouped in category 1), *de facto* leading to a loss of information. Nevertheless, this loss is limited as the difference between sufficient diagnostic quality (*i*.*e*. a grade greater or equal to 3) and insufficient examinations (grade ≤2) remains. One can also point out the relatively small number of patients as a limitation. However, the use of a Bayesian inference analysis, with simulation of large population derived from this sample, gives us sufficient statistical power to demonstrate the equivalence in LV assessment. Another limitation is the only moderate agreement between the two radiologists. This result needs to be put into perspective with (i) the number of categories -four- of our evaluation: the greater number of categories, the lower the concordance, (ii) the fact that the disagreement was, in the vast majority of cases, between class 3 and 4 (86% of disagreements for the overall image quality assessment, for example) which does not impact on the diagnosis or work-up of patients. One last limitation is that this study is vendor-specific, and limited to 3T scanner with dual-source RF transmission technology. It is, however, more than likely that our results could be applied to any 3T scanner.

## Conclusion

We demonstrated that in 3T cardiac magnetic resonance imaging with dual-source RF transmission technology, the acquisition of b-TFE cine sequences after gadolinium injection does not lead to any significant differences in the qualitative and quantitative assessment of left ventricular ejection fraction, mass and volume, despite a significantly deteriorated overall image quality.

## Supporting Information

S1 Dataset Statistical analyses. (PDF)

## Abbreviations

CMR: Cardiac magnetic resonance
CNR: Contrast to Noise Ratio
LV: Left ventricle
LVEF: Left ventricular ejection fraction
ROI: Region of interest
SAR: Specific Absorption Rate
1.5T—3T: 1.5 Tesla—3 Teslas
