# Supplementary material for: Left Ventricular Function Evaluation on a 3T MR Scanner with Parallel RF Transmission Technique: Prospective Comparison of Cine Sequences Acquired before and after Gadolinium Injection
Source: PLoS One. 2016 Sep 26;11(9):e0163503. doi: 10.1371/journal.pone.0163503 (PMC5036819; doi:10.1371/journal.pone.0163503)
Supplement: S1 Dataset — (PDF) [file pone.0163503.s001.pdf]

Analyses : POST–GADO

9 Comparaisons pré-post pour les variables quantiatives

Décrivons dans un premier temps les variables quantitatives pré et post confondus, puis en distinguant les deux périodes :

Variable	n	Min	q_1	$\widetilde{x}$	$\bar{x}$	q_3	Max	s	IQR	#NA
FEVG	50	18.0	44.2	53.0	52.4	66.5	77.0	16.5	22.2	0
VTD	50	92.0	121.5	138.5	162.3	207.2	305.0	58.7	85.8	0
Masse	50	54.0	92.5	124.5	129.5	162.5	245.0	44.3	70.0	0
ContrastBrui	50	2.7	10.3	16.2	17.4	23.4	67.9	10.7	13.1	0

Table 7 – Descriptif sur les deux périodes confondues

Variable	Levels	n	Min	q_1	$\widetilde{x}$	$\bar{x}$	q_3	Max	s	IQR	#NA
FEVG	1	25	18.0	44.0	53.0	52.6	67.0	77.0	16.4	23.0	0
	2	25	19.0	45.0	54.0	52.2	64.0	77.0	16.9	19.0	0
	all	50	18.0	44.2	53.0	52.4	66.5	77.0	16.5	22.2	0
VTD	1	25	92.0	126.0	137.0	162.8	199.0	305.0	60.7	73.0	0
	2	25	93.0	121.0	140.0	161.8	210.0	279.0	57.9	89.0	0
	all	50	92.0	121.5	138.5	162.3	207.2	305.0	58.7	85.8	0
Masse	1	25	54.0	90.0	119.0	128.2	158.0	245.0	46.8	68.0	0
	2	25	60.0	97.0	134.0	130.8	164.0	210.0	42.6	67.0	0
	all	50	54.0	92.5	124.5	129.5	162.5	245.0	44.3	70.0	0
ContrastBrui	1	25	6.1	10.6	19.1	19.7	23.4	67.9	13.1	12.9	0
	2	25	2.7	9.2	14.6	15.2	19.9	28.3	7.3	10.7	0
	all	50	2.7	10.3	16.2	17.4	23.4	67.9	10.7	13.1	0

Table 8 – Analyses descriptives des variables quantitatives en distinguant les deux périodes

10 FEVG

	mean	sd	2.5%	25%	50%	75%	97.5%
b0	52.7505	3.1916	46.3900	50.7000	52.7400	54.8400	59.0700
bTPS[2]	-0.3230	0.6600	-1.6250	-0.7563	-0.3203	0.1075	0.9815
diffT2T1	-0.3230	0.6600	-1.6250	-0.7563	-0.3203	0.1075	0.9815
PrdiffT2T1	0.3072	0.4613	0.0000	0.0000	0.0000	1.0000	1.0000
deviance	224.3388	10.8822	206.2000	216.6000	223.3000	231.0000	248.3025

11 VTD

	mean	sd	2.5%	25%	50%	75%	97.5%
b0	5.0368	0.0883	4.8620	4.9790	5.0370	5.0950	5.2120
bTPS[2]	-0.0032	0.0312	-0.0645	-0.0237	-0.0030	0.0172	0.0584
diffT2T1	-0.0032	0.0312	-0.0645	-0.0237	-0.0030	0.0172	0.0584
PrdiffT2T1	0.4604	0.4984	0.0000	0.0000	0.0000	1.0000	1.0000
deviance	-98.6884	14.5301	-124.8000	-108.9000	-99.5000	-89.3400	-68.2700

12 Masse

	mean	sd	2.5%	25%	50%	75%	97.5%
b0	128.3756	8.7966	110.9000	122.6000	128.5000	134.2000	145.5000
bTPS[2]	2.5500	4.2427	-5.8291	-0.2300	2.5720	5.3200	10.9400
diffT2T1	2.5500	4.2427	-5.8291	-0.2300	2.5720	5.3200	10.9400
PrdiffT2T1	0.7319	0.4430	0.0000	0.0000	1.0000	1.0000	1.0000
deviance	410.4729	11.0870	392.0000	402.6000	409.4000	417.2000	435.1000

13 Contraste bruit

	mean	sd	2.5%	25%	50%	75%	97.5%
b0	2.8104	0.1419	2.5300	2.7170	2.8110	2.9050	3.0860
bTPS[2]	-0.2260	0.1357	-0.4930	-0.3156	-0.2253	-0.1369	0.0425
diffT2T1	-0.2260	0.1357	-0.4930	-0.3156	-0.2253	-0.1369	0.0425
PrdiffT2T1	0.0473	0.2123	0.0000	0.0000	0.0000	0.0000	1.0000
deviance	65.8107	8.5513	50.6997	59.7300	65.2900	71.2900	83.9700

14 Comparaisons pré-post pour les variables qualitatives

Présentons dans un premier temps les analyses descriptives en confondant les lecteurs avant et en les confondants après :

**Variable**	**Levels**	**n**	%	$\sum$ %
QualiteGlobale01	0	11	11.0	11.0
	1	89	89.0	100.0
	all	100	100.0	
Contours01	0	12	12.0	12.0
	1	88	88.0	100.0
	all	100	100.0	
Artefacts01	0	54	54.0	54.0
	1	46	46.0	100.0
	all	100	100.0	
EvalCine01	0	2	2.0	2.0
	1	98	98.0	100.0
	all	100	100.0	

Table 9 – Descriptif global, temps et lecteurs confondus

**Variable**	**Levels**	$\mathbf{n}_1$	$\%_1$	$\sum \%_1$	$\mathbf{n}_2$	$\%_2$	$\sum \%_2$	$\mathbf{n}_{\text{all}}$	$\%_{\text{all}}$	$\sum \%_{\text{all}}$
QualiteGlobale01	0	3	6.0	6.0	8	16.0	16.0	11	11.0	11.0
	1	47	94.0	100.0	42	84.0	100.0	89	89.0	100.0
	all	50	100.0		50	100.0		100	100.0	
Contours01	0	3	6.0	6.0	9	18.0	18.0	12	12.0	12.0
	1	47	94.0	100.0	41	82.0	100.0	88	88.0	100.0
	all	50	100.0		50	100.0		100	100.0	
Artefacts01	0	26	52.0	52.0	28	56.0	56.0	54	54.0	54.0
	1	24	48.0	100.0	22	44.0	100.0	46	46.0	100.0
	all	50	100.0		50	100.0		100	100.0	
EvalCine01	0	0	0.0	0.0	2	4.0	4.0	2	2.0	2.0
	1	50	100.0	100.0	48	96.0	100.0	98	98.0	100.0
	all	50	100.0		50	100.0		100	100.0	

Table 10 – Descriptif en distinguant avant-après, lecteurs confondus

15 Variable Qualité Globale

	mean	sd	2.5%	25%	50%	75%	97.5%
b0	4.5822	1.5246	1.5970	3.5947	4.5330	5.5430	7.6371
bTPS[2]	-1.7463	0.9601	-3.7860	-2.3360	-1.6900	-1.0948	-0.0305
diffT2T1	-1.7463	0.9601	-3.7860	-2.3360	-1.6900	-1.0948	-0.0305
PrdiffT2T1	0.0228	0.1493	0.0000	0.0000	0.0000	0.0000	0.0000
deviance	33.1405	7.0587	21.5497	28.0800	32.4700	37.4000	48.7605

16 Variable Contours

	mean	sd	2.5%	25%	50%	75%	97.5%
b0	4.8552	1.6896	1.5340	3.7957	4.8180	5.8930	8.3662
bTPS[2]	-2.1224	1.0145	-4.2950	-2.7752	-2.0580	-1.4240	-0.2973
diffT2T1	-2.1224	1.0145	-4.2950	-2.7752	-2.0580	-1.4240	-0.2973
PrdiffT2T1	0.0092	0.0955	0.0000	0.0000	0.0000	0.0000	0.0000
deviance	31.1374	7.0466	19.6100	26.0800	30.3100	35.4000	46.8307

17 Variable Artefacts

	mean	sd	2.5%	25%	50%	75%	97.5%
b0	-0.0831	1.4323	-2.9901	-0.8743	-0.0742	0.7703	2.6190
bTPS[2]	-0.3037	0.5502	-1.3960	-0.6734	-0.2982	0.0663	0.7615
diffT2T1	-0.3037	0.5502	-1.3960	-0.6734	-0.2982	0.0663	0.7615
PrdiffT2T1	0.2924	0.4549	0.0000	0.0000	0.0000	1.0000	1.0000
deviance	91.7474	3.4563	86.3700	89.2500	91.3200	93.6900	99.7000

18 Variable Eval Cine

	mean	sd	2.5%	25%	50%	75%	97.5%
b0	4.9424	1.8634	0.5825	3.9478	4.9865	6.0323	8.5812
bTPS[2]	-1.4670	1.4451	-4.7180	-2.3405	-1.4115	-0.4795	1.1271
diffT2T1	-1.4670	1.4451	-4.7180	-2.3405	-1.4115	-0.4795	1.1271
PrdiffT2T1	0.1483	0.3555	0.0000	0.0000	0.0000	0.0000	1.0000
deviance	15.9319	3.7091	8.7950	13.5600	15.7700	18.1700	24.2705

Quatrième partie

Analyses sous-groupes

Notations :

Codage dans le modèle	Groupe
1	PathologiesIschemiques
2	Myocardite
3	cardiomyopathie

19 Etude de la variable Qualité globale en fonction du sous-groupe

	mean	sd	2.5%	25%	50%	75%	97.5%
b0	3.9487	1.2208	1.4320	3.2120	3.9710	4.7270	6.2960
bTPS[2]	-3.2396	0.7432	-4.7760	-3.7240	-3.2070	-2.7200	-1.8860
bTPS[3]	0.7703	0.8371	-0.8184	0.1978	0.7521	1.3150	2.4770
bGRP[2]	2.1528	2.0618	-1.6970	0.7247	2.0880	3.5010	6.3650
bGRP[3]	-1.9069	1.0561	-4.0260	-2.6040	-1.8870	-1.1987	0.1336
bGRPTPS[2,2]	-0.0773	2.0513	-4.2240	-1.4292	-0.0345	1.3190	3.8370
bGRPTPS[2,3]	0.9088	2.7071	-4.0580	-0.9835	0.7952	2.6800	6.5120
bGRPTPS[3,2]	1.7796	0.9680	-0.0887	1.1250	1.7670	2.4260	3.6980
bGRPTPS[3,3]	0.1305	1.1107	-2.0640	-0.6089	0.1371	0.8727	2.2980
diff G1 T2T1	-3.2396	0.7432	-4.7760	-3.7240	-3.2070	-2.7200	-1.8860
diff G1 T3T1	0.7703	0.8371	-0.8184	0.1978	0.7521	1.3150	2.4770
diff G1 T3T2	4.0100	0.9096	2.3820	3.3760	3.9580	4.5860	5.9320
diff G2 T2T1	-3.3169	2.0910	-7.5900	-4.6830	-3.2490	-1.8790	0.5818
diff G2 T3T1	1.6791	2.7842	-3.4140	-0.2615	1.5500	3.4932	7.4880
diff G2 T3T2	4.9960	3.1753	-0.5298	2.7270	4.7570	7.0520	11.7800
diff G3 T2T1	-1.4600	0.7353	-2.9560	-1.9410	-1.4450	-0.9618	-0.0645
diff G3 T3T1	0.9008	0.8104	-0.6396	0.3519	0.8873	1.4320	2.5330
diff G3 T3T2	2.3608	0.8429	0.7947	1.7800	2.3280	2.9090	4.0990
diff T1 G2G1	2.1528	2.0618	-1.6970	0.7247	2.0880	3.5010	6.3650
diff T1 G3G1	-1.9069	1.0561	-4.0260	-2.6040	-1.8870	-1.1987	0.1336
diff T1 G3G2	-4.0597	2.2285	-8.6120	-5.5230	-3.9895	-2.5200	0.1059
diff T2 G2G1	2.0755	1.8517	-1.3460	0.8276	1.9910	3.2380	5.9700
diff T2 G3G1	-0.1273	0.9663	-2.0000	-0.7692	-0.1343	0.5035	1.8090
diff T2 G3G2	-2.2028	1.9699	-6.2810	-3.4500	-2.1330	-0.8862	1.5020
diff T3 G2G1	3.0615	3.1651	-2.5520	0.8032	2.8560	5.1190	9.7860
diff T3 G3G1	-1.7765	1.2500	-4.2940	-2.5860	-1.7590	-0.9393	0.6316
diff T3 G3G2	-4.8380	3.3033	-11.8800	-6.9880	-4.6240	-2.4850	1.0010
PRdiff G1 T2T1	0.0000						
PRdiff G1 T3T1	0.8220						
PRdiff G1 T3T2	1.0000						
PRdiff G2 T2T1	0.0497						
PRdiff G2 T3T1	0.7173						
PRdiff G2 T3T2	0.9583						
PRdiff G3 T2T1	0.0198						
PRdiff G3 T3T1	0.8694						
PRdiff G3 T3T2	0.9988						
PRdiff T1 G2G1	0.8530						
PRdiff T1 G3G1	0.0335						
PRdiff T1 G3G2	0.0281						
PRdiff T2 G2G1	0.8774						
PRdiff T2 G3G1	0.4415						
PRdiff T2 G3G2	0.1244						
PRdiff T3 G2G1	0.8311						
PRdiff T3 G3G1	0.0737						
PRdiff T3 G3G2	0.0581						
deviance	157.7827	11.7336	137.1000	149.5000	157.0000	165.2000	182.8000

20 Etude de la variable Artefacts en fonction du sous-groupe

	mean	sd	2.5%	25%	50%	75%	97.5%
b0	3.1831	1.1285	0.7149	2.5320	3.2210	3.8910	5.3230
bTPS[2]	-3.1414	0.6618	-4.5110	-3.5720	-3.1150	-2.6860	-1.9130
bTPS[3]	-0.1867	0.6434	-1.4460	-0.6201	-0.1866	0.2453	1.0850
bGRP[2]	-0.3034	1.6390	-3.4730	-1.4020	-0.3306	0.7762	2.9930
bGRP[3]	-2.0873	1.0919	-4.2870	-2.7960	-2.0720	-1.3560	0.0226
bGRPTPS[2,2]	0.0657	1.4457	-2.8890	-0.8824	0.1089	1.0520	2.8070
bGRPTPS[2,3]	0.1302	1.6148	-2.9850	-0.9470	0.1041	1.1790	3.3870
bGRPTPS[3,2]	1.7829	0.9469	-0.0572	1.1440	1.7730	2.4170	3.6610
bGRPTPS[3,3]	0.3663	0.9692	-1.5330	-0.2871	0.3670	1.0160	2.2690
diff G1 T2T1	-3.1414	0.6618	-4.5110	-3.5720	-3.1150	-2.6860	-1.9130
diff G1 T3T1	-0.1867	0.6434	-1.4460	-0.6201	-0.1866	0.2453	1.0850
diff G1 T3T2	2.9546	0.6873	1.6870	2.4790	2.9270	3.3980	4.3910
diff G2 T2T1	-3.0757	1.3971	-5.9910	-3.9800	-3.0170	-2.1060	-0.4907
diff G2 T3T1	-0.0566	1.5700	-3.0790	-1.1010	-0.0789	0.9581	3.1160
diff G2 T3T2	3.0192	1.5764	0.2123	1.9280	2.9160	3.9980	6.4090
diff G3 T2T1	-1.3585	0.7666	-2.9110	-1.8650	-1.3430	-0.8388	0.1045
diff G3 T3T1	0.1796	0.7715	-1.3260	-0.3402	0.1761	0.6966	1.7020
diff G3 T3T2	1.5381	0.8064	0.0126	0.9888	1.5170	2.0650	3.1850
diff T1 G2G1	-0.3034	1.6390	-3.4730	-1.4020	-0.3306	0.7762	2.9930
diff T1 G3G1	-2.0873	1.0919	-4.2870	-2.7960	-2.0720	-1.3560	0.0226
diff T1 G3G2	-1.7839	1.8113	-5.4550	-2.9680	-1.7580	-0.5601	1.6790
diff T2 G2G1	-0.2377	1.5967	-3.3430	-1.3050	-0.2464	0.8164	2.9440
diff T2 G3G1	-0.3044	1.0664	-2.3950	-1.0082	-0.3089	0.3912	1.8190
diff T2 G3G2	-0.0667	1.7479	-3.5390	-1.2180	-0.0614	1.0880	3.3750
diff T3 G2G1	-0.1732	1.8872	-3.7640	-1.4390	-0.2116	1.0450	3.6540
diff T3 G3G1	-1.7210	1.1557	-4.0220	-2.4800	-1.7140	-0.9575	0.5446
diff T3 G3G2	-1.5478	2.0089	-5.6570	-2.8520	-1.4980	-0.1932	2.2910
PRdiff G1 T2T1	0.0000						
PRdiff G1 T3T1	0.3840						
PRdiff G1 T3T2	1.0000						
PRdiff G2 T2T1	0.0088						
PRdiff G2 T3T1	0.4796						
PRdiff G2 T3T2	0.9833						
PRdiff G3 T2T1	0.0346						
PRdiff G3 T3T1	0.5910						
PRdiff G3 T3T2	0.9760						
PRdiff T1 G2G1	0.4195						
PRdiff T1 G3G1	0.0261						
PRdiff T1 G3G2	0.1600						
PRdiff T2 G2G1	0.4373						
PRdiff T2 G3G1	0.3825						
PRdiff T2 G3G2	0.4852						
PRdiff T3 G2G1	0.4541						
PRdiff T3 G3G1	0.0655						
PRdiff T3 G3G2	0.2186						
deviance	193.4011	11.0967	173.6000	185.6000	192.7000	200.5000	217.0000

21 Etude de la variable Contrastes Contour en fonction du sous-groupe

	mean	sd	2.5%	25%	50%	75%	97.5%
b0	2.1746	1.0334	-0.0412	1.5960	2.2130	2.7990	4.1430
bTPS[2]	-1.5146	0.5028	-2.5340	-1.8440	-1.5060	-1.1730	-0.5521
bTPS[3]	0.6594	0.5957	-0.4958	0.2581	0.6514	1.0520	1.8500
bGRP[2]	0.4299	1.2939	-2.0120	-0.4495	0.3916	1.2750	3.0750
bGRP[3]	-1.5702	0.7146	-3.0050	-2.0460	-1.5600	-1.0840	-0.1995
bGRPTPS[2,2]	0.3280	1.4079	-2.4770	-0.5985	0.3341	1.2720	3.0650
bGRPTPS[2,3]	-0.4084	1.6125	-3.4890	-1.5000	-0.4460	0.6304	2.8890
bGRPTPS[3,2]	0.5398	0.7612	-0.9481	0.0278	0.5395	1.0460	2.0450
bGRPTPS[3,3]	-0.0778	0.8454	-1.7360	-0.6457	-0.0817	0.4908	1.5830
diff G1 T2T1	-1.5146	0.5028	-2.5340	-1.8440	-1.5060	-1.1730	-0.5521
diff G1 T3T1	0.6594	0.5957	-0.4958	0.2581	0.6514	1.0520	1.8500
diff G1 T3T2	2.1740	0.5856	1.0770	1.7710	2.1570	2.5570	3.3700
diff G2 T2T1	-1.1866	1.3766	-3.9480	-2.0860	-1.1650	-0.2656	1.4760
diff G2 T3T1	0.2510	1.5694	-2.7160	-0.8039	0.2108	1.2530	3.4820
diff G2 T3T2	1.4376	1.6075	-1.5440	0.3566	1.3700	2.4460	4.8190
diff G3 T2T1	-0.9748	0.6055	-2.1740	-1.3780	-0.9683	-0.5675	0.2034
diff G3 T3T1	0.5816	0.6294	-0.6384	0.1571	0.5754	1.0010	1.8350
diff G3 T3T2	1.5564	0.6460	0.3299	1.1168	1.5450	1.9800	2.8550
diff T1 G2G1	0.4299	1.2939	-2.0120	-0.4495	0.3916	1.2750	3.0750
diff T1 G3G1	-1.5702	0.7146	-3.0050	-2.0460	-1.5600	-1.0840	-0.1995
diff T1 G3G2	-2.0001	1.3835	-4.8490	-2.9010	-1.9500	-1.0480	0.5793
diff T2 G2G1	0.7579	1.2574	-1.6230	-0.0932	0.7238	1.5700	3.3310
diff T2 G3G1	-1.0304	0.6900	-2.4050	-1.4810	-1.0220	-0.5689	0.3156
diff T2 G3G2	-1.7883	1.3314	-4.5260	-2.6480	-1.7450	-0.8883	0.7267
diff T3 G2G1	0.0215	1.5578	-2.8160	-1.0380	-0.0661	0.9918	3.3370
diff T3 G3G1	-1.6480	0.8114	-3.2740	-2.1840	-1.6330	-1.1030	-0.0886
diff T3 G3G2	-1.6695	1.6114	-5.1050	-2.6780	-1.5840	-0.5608	1.2530
PRdiff G1 T2T1	0.0009						
PRdiff G1 T3T1	0.8688						
PRdiff G1 T3T2	0.9999						
PRdiff G2 T2T1	0.1918						
PRdiff G2 T3T1	0.5544						
PRdiff G2 T3T2	0.8199						
PRdiff G3 T2T1	0.0520						
PRdiff G3 T3T1	0.8227						
PRdiff G3 T3T2	0.9937						
PRdiff T1 G2G1	0.6215						
PRdiff T1 G3G1	0.0120						
PRdiff T1 G3G2	0.0669						
PRdiff T2 G2G1	0.7258						
PRdiff T2 G3G1	0.0654						
PRdiff T2 G3G2	0.0822						
PRdiff T3 G2G1	0.4828						
PRdiff T3 G3G1	0.0192						
PRdiff T3 G3G2	0.1424						
deviance	251.0523	11.4918	230.6000	242.9000	250.4000	258.4000	275.4000

22 Etude de la variable Rapport Contrastes Bruit en fonction du sous-groupe

	mean	sd	2.5%	25%	50%	75%	97.5%
b0	2.2401	0.1210	2.0060	2.1590	2.2370	2.3210	2.4835
bTPS[2]	-0.9218	0.1268	-1.1665	-1.0090	-0.9203	-0.8362	-0.6796
bTPS[3]	0.0775	0.1279	-0.1712	-0.0092	0.0772	0.1617	0.3318
bGRP[2]	-0.6028	0.3471	-1.2890	-0.8343	-0.6009	-0.3704	0.0684
bGRP[3]	-0.1663	0.2075	-0.5803	-0.3031	-0.1595	-0.0303	0.2365
bGRPTPS[2,2]	0.0846	0.3700	-0.6541	-0.1580	0.0844	0.3331	0.8130
bGRPTPS[2,3]	-0.1013	0.3770	-0.8388	-0.3547	-0.1051	0.1507	0.6427
bGRPTPS[3,2]	-0.0528	0.2175	-0.4752	-0.1997	-0.0517	0.0925	0.3800
bGRPTPS[3,3]	-0.1305	0.2206	-0.5728	-0.2752	-0.1268	0.0182	0.2989
diff G1 T2T1	-0.9218	0.1268	-1.1665	-1.0090	-0.9203	-0.8362	-0.6796
diff G1 T3T1	0.0775	0.1279	-0.1712	-0.0092	0.0772	0.1617	0.3318
diff G1 T3T2	0.9993	0.1276	0.7383	0.9142	0.9995	1.0840	1.2480
diff G2 T2T1	-0.8372	0.3510	-1.5326	-1.0700	-0.8421	-0.6053	-0.1318
diff G2 T3T1	-0.0239	0.3552	-0.7265	-0.2631	-0.0275	0.2190	0.6648
diff G2 T3T2	0.8134	0.3567	0.1143	0.5777	0.8107	1.0560	1.5091
diff G3 T2T1	-0.9746	0.1816	-1.3251	-1.0950	-0.9756	-0.8536	-0.6214
diff G3 T3T1	-0.0530	0.1835	-0.4113	-0.1742	-0.0531	0.0702	0.3079
diff G3 T3T2	0.9216	0.1798	0.5606	0.8008	0.9206	1.0440	1.2730
diff T1 G2G1	-0.6028	0.3471	-1.2890	-0.8343	-0.6009	-0.3704	0.0684
diff T1 G3G1	-0.1663	0.2075	-0.5803	-0.3031	-0.1595	-0.0303	0.2365
diff T1 G3G2	0.4365	0.3805	-0.3104	0.1819	0.4370	0.6816	1.1955
diff T2 G2G1	-0.5182	0.3533	-1.2075	-0.7598	-0.5160	-0.2785	0.1626
diff T2 G3G1	-0.2191	0.2074	-0.6311	-0.3561	-0.2165	-0.0807	0.1843
diff T2 G3G2	0.2991	0.3756	-0.4199	0.0465	0.2998	0.5490	1.0390
diff T3 G2G1	-0.7042	0.3597	-1.4195	-0.9426	-0.7087	-0.4595	0.0051
diff T3 G3G1	-0.2968	0.2138	-0.7291	-0.4396	-0.2913	-0.1542	0.1122
diff T3 G3G2	0.4073	0.3766	-0.3263	0.1562	0.4046	0.6599	1.1585
PRdiff G1 T2T1	0.0000						
PRdiff G1 T3T1	0.7278						
PRdiff G1 T3T2	1.0000						
PRdiff G2 T2T1	0.0091						
PRdiff G2 T3T1	0.4689						
PRdiff G2 T3T2	0.9889						
PRdiff G3 T2T1	0.0000						
PRdiff G3 T3T1	0.3858						
PRdiff G3 T3T2	1.0000						
PRdiff T1 G2G1	0.0398						
PRdiff T1 G3G1	0.2064						
PRdiff T1 G3G2	0.8749						
PRdiff T2 G2G1	0.0720						
PRdiff T2 G3G1	0.1369						
PRdiff T2 G3G2	0.7882						
PRdiff T3 G2G1	0.0258						
PRdiff T3 G3G1	0.0800						
PRdiff T3 G3G2	0.8596						
deviance	212.4591	12.2484	189.9475	203.9750	211.8000	220.3000	238.2000

23 Etude de la variable Surface Bruit en fonction du sous-groupe

	mean	sd	2.5%	25%	50%	75%	97.5%
b0	0.3402	0.1714	0.0070	0.2251	0.3390	0.4540	0.6819
bTPS[2]	-0.0561	0.0627	-0.1798	-0.0980	-0.0560	-0.0142	0.0673
bTPS[3]	0.1318	0.0629	0.0085	0.0895	0.1318	0.1739	0.2557
bGRP[2]	-1.3729	0.5061	-2.3730	-1.7090	-1.3720	-1.0430	-0.3599
bGRP[3]	-0.6104	0.2948	-1.1920	-0.8077	-0.6088	-0.4119	-0.0384
bGRPTPS[2,2]	-0.0147	0.1847	-0.3770	-0.1380	-0.0154	0.1086	0.3496
bGRPTPS[2,3]	0.0550	0.1850	-0.3095	-0.0678	0.0549	0.1787	0.4164
bGRPTPS[3,2]	0.0472	0.1093	-0.1659	-0.0266	0.0467	0.1206	0.2623
bGRPTPS[3,3]	-0.0057	0.1094	-0.2203	-0.0791	-0.0058	0.0678	0.2091
diff G1 T2T1	-0.0561	0.0627	-0.1798	-0.0980	-0.0560	-0.0142	0.0673
diff G1 T3T1	0.1318	0.0629	0.0085	0.0895	0.1318	0.1739	0.2557
diff G1 T3T2	0.1879	0.0626	0.0647	0.1458	0.1881	0.2300	0.3109
diff G2 T2T1	-0.0708	0.1736	-0.4128	-0.1870	-0.0714	0.0454	0.2710
diff G2 T3T1	0.1869	0.1744	-0.1569	0.0706	0.1866	0.3041	0.5286
diff G2 T3T2	0.2577	0.1744	-0.0840	0.1411	0.2579	0.3746	0.5983
diff G3 T2T1	-0.0088	0.0900	-0.1854	-0.0692	-0.0087	0.0512	0.1674
diff G3 T3T1	0.1261	0.0899	-0.0494	0.0659	0.1263	0.1862	0.3034
diff G3 T3T2	0.1349	0.0902	-0.0423	0.0745	0.1348	0.1955	0.3116
diff T1 G2G1	-1.3729	0.5061	-2.3730	-1.7090	-1.3720	-1.0430	-0.3599
diff T1 G3G1	-0.6104	0.2948	-1.1920	-0.8077	-0.6088	-0.4119	-0.0384
diff T1 G3G2	0.7625	0.5324	-0.2890	0.4107	0.7589	1.1200	1.8060
diff T2 G2G1	-1.3877	0.5072	-2.3870	-1.7240	-1.3880	-1.0540	-0.3785
diff T2 G3G1	-0.5632	0.2943	-1.1410	-0.7598	-0.5624	-0.3655	0.0160
diff T2 G3G2	0.8245	0.5345	-0.2254	0.4713	0.8207	1.1820	1.8790
diff T3 G2G1	-1.3179	0.5079	-2.3190	-1.6550	-1.3170	-0.9857	-0.3060
diff T3 G3G1	-0.6162	0.2942	-1.1960	-0.8123	-0.6162	-0.4196	-0.0384
diff T3 G3G2	0.7017	0.5344	-0.3504	0.3471	0.6999	1.0590	1.7550
PRdiff G1 T2T1	0.1846						
PRdiff G1 T3T1	0.9821						
PRdiff G1 T3T2	0.9984						
PRdiff G2 T2T1	0.3409						
PRdiff G2 T3T1	0.8591						
PRdiff G2 T3T2	0.9303						
PRdiff G3 T2T1	0.4612						
PRdiff G3 T3T1	0.9194						
PRdiff G3 T3T2	0.9337						
PRdiff T1 G2G1	0.0037						
PRdiff T1 G3G1	0.0183						
PRdiff T1 G3G2	0.9238						
PRdiff T2 G2G1	0.0039						
PRdiff T2 G3G1	0.0279						
PRdiff T2 G3G2	0.9388						
PRdiff T3 G2G1	0.0054						
PRdiff T3 G3G1	0.0184						
PRdiff T3 G3G2	0.9069						
deviance	-26.6630	16.6238	-56.8200	-38.3000	-27.4900	-15.9500	7.9921
